# Genomic Insights Into Selected *Mycobacterium orygis* and *Mycobacterium tuberculosis* Isolates From Captive Wild and Domestic Animals of India: Challenge Posed for TB Elimination Programs

**DOI:** 10.1155/pm/6264505

**Published:** 2026-11-15

**Authors:** Payal Soni, Anvita Gupta Malhotra, Satyaprakash Vishwakarma, Mahesh Samantaray, Manoj Kumar, Anand Kumar Maurya, Jitendra Singh, Sarman Singh

**Affiliations:** ^1^ Department of Microbiology All India Institute of Medical Sciences Bhopal Madhya Pradesh India aiims.edu; ^2^ Department of Translational Medicine All India Institute of Medical Sciences Bhopal Madhya Pradesh India aiims.edu; ^3^ Department of Veterinary Microbiology Bihar Veterinary College Patna India; ^4^ Advanced Center for Chronic and Rare Diseases South Asian University Road New Delhi India

## Abstract

**Background:**

Tuberculosis caused by members of the *Mycobacterium tuberculosis* complex (MTBC) poses significant threats to animal health, public health, and wildlife conservation. *Mycobacterium orygis*, an emerging MTBC member, remains poorly characterized with respect to its epidemiology and transmission dynamics among Indian wildlife and livestock.

**Methods:**

Fifty‐five necropsy tissue samples from 21 captive wild animals were subjected to mycobacterial culture and a panel of molecular diagnostic assays, including multiplex PCR, Xpert‐Ultra, and Truenat MTB/Rif. Confirmed MTBC isolates were characterized by drug susceptibility testing (GenoType MTBDRplus) and spoligotyping. Six isolates were selected for whole‐genome sequencing (WGS), and phylogenetic analyses were performed against publicly available reference genomes.

**Results:**

Of 55 samples, 38 were MTBC‐positive and 6 yielded nontuberculous mycobacteria. Drug susceptibility profiling revealed 28 pan‐susceptible isolates, nine with isoniazid monoresistance, and one with rifampicin monoresistance. Spoligotyping classified 10 isolates as CAS1‐Delhi, 27 as orphan lineages, and one remained unassigned. WGS of six isolates, representing diverse host species and tissue types, identified four as *M. tuberculosis* (CAS1‐Delhi lineage). Notably, one orphan isolate from a blackbuck was reclassified as *M. orygis*. Phylogenetic analysis revealed that this *M. orygis* strain was genetically distinct from other Indian wildlife strains and most closely related to a human‐derived isolate from Karnataka, suggesting cross‐host genomic relatedness. The remaining isolates clustered with Indian and global reference genomes; one was closely related to cattle‐derived strains, whereas four were more closely related to human‐derived strains. Low pairwise SNP distances (0–78 SNPs) with strong bootstrap support indicated close genomic relatedness across host species and geographic regions.

**Conclusion:**

This study shows the MTBC genomic diversity among various hosts. The phylogenetic relationships suggest that there is genetic relatedness among host populations such as *M. orygis* in a captive Indian blackbuck, which was in human contact for a long time. The study demonstrates the importance of integrated One Health genomic surveillance of mycobacterial infections at the human‐animal interface and calls for more extensive, epidemiologically linked studies to look for cross‐host transmission pathways.

## 1. Introduction

Tuberculosis (TB) is a chronic, persistent, and highly infectious disease caused by members of the *Mycobacterium tuberculosis* complex (MTBC), and poses substantial risks to livestock, wildlife, and human health. The MTBC encompasses *M. tuberculosis* and nine closely related species: *Mycobacterium bovis*, *Mycobacterium africanum*, *Mycobacterium caprae*, *Mycobacterium pinnipedii*, *Mycobacterium microti*, *Mycobacterium mungi*, *Mycobacterium canettii*, *Mycobacterium orygis*, and *Mycobacterium suricattae* [1]. With a broad host range and significant zoonotic potential, MTBC members have been documented in more than 40 free‐ranging wild animal species. These wildlife hosts may function either as “maintenance hosts,” sustaining infection independently within a population, or as “spillover hosts,” where transmission depends on continued exposure from an external reservoir [2].

Although *M. bovis* has historically been recognized as the principal causative agent of animal TB, *M. orygis* has emerged as an increasingly significant pathogen in both humans and animals, particularly South and Southeast Asian countries including India, Bangladesh, Nepal, and Pakistan [3, 4]. This species has been documented across a diverse range of hosts, including cattle, African buffalo, blackbuck, spotted deer, bison, rhesus monkeys, rhinoceros, and humans in both captive and free‐ranging settings. Despite this broad host range, its biology, genomic characteristics, and epidemiology remain incompletely understood. The identity of its primary reservoir host continues to be debated. Although *M. orygis* was historically regarded as a wildlife‐associated pathogen, emerging evidence suggests that cattle may serve as the principal reservoir, with spillover events driving transmission to wildlife and humans. In India, infections have been documented in humans and domestic animals, with sporadic reports in wildlife further emphasizing the need for cross‐species surveillance [4, 5].

Accurate disease diagnosis is a cornerstone of effective TB control, yet it remains a formidable challenge in wildlife populations. Reliable diagnostic tools are essential not only for case detection but also for evaluating surveillance programs, characterizing transmission pathways, and advancing our understanding of host–pathogen interactions. Improving diagnostic capacity across species is therefore a critical step toward addressing TB at the human–animal–environment interface within a One Health framework [6, 7].

Traditionally, genotyping of MTBC isolates has played a central role in distinguishing recent transmission from disease recurrence, identifying epidemiologically linked clusters, and elucidating pathogen evolution and lineage‐specific transmission patterns. Among available genotyping methods, spacer oligonucleotide typing (spoligotyping) remains widely employed in molecular epidemiology and TB surveillance. However, spoligotyping has important limitations: it does not reliably differentiate all MTBC species or lineages, particularly less common members of the MTBC, such as *M. orygis*, underscoring the need for more robust and discriminatory molecular approaches [8].

The advent of whole‐genome sequencing (WGS) has transformed infectious disease diagnostics and epidemiology. Unlike targeted sequencing approaches, which yield comparatively low‐resolution data, WGS interrogates virtually the entire genome, offering markedly superior discriminatory power [9, 10]. Within the MTBC, WGS enables precise species and strain differentiation, prediction of antimicrobial resistance, and high‐resolution insight into transmission dynamics. It also facilitates the detection of mixed infections, distinction between relapse and reinfection, and investigation of potential zoonotic origins or cross‐border transmission events [4, 11, 12].

In a prior study, we reported the microbiological identification and preliminary molecular characterization of MTBC isolates recovered from captive wild animals using targeted genotyping [13]. Although that approach provided a foundational understanding, it offered limited resolution for precise lineage assignment and comprehensive genomic characterization. In the present study, we performed WGS of six representative isolates to achieve higher‐resolution lineage assignment, reclassify a previously unresolved orphan isolate as *M. orygis*, and conduct robust comparative phylogenomic analyses to elucidate the genomic relatedness of these isolates with previously reported MTBC genomes from diverse host species and geographic regions.

## 2. Materials and Methods

### 2.1. Sample Collection

Necropsy tissue samples were collected from deceased animals suspected of having TB from a zoological park in Patna, India, as reported elsewhere [13].

These samples were obtained from different captive wild animal hosts, including barking deer, black bear, blackbuck, blue bull, lioness, peacock, one‐horned rhinoceros, sloth bear, spotted deer, swamp deer, tigress, and wolf cub. This research was conducted as a cross‐sectional study in which all available postmortem necropsy tissue samples from suspected TB cases were collected and analyzed at a single point in time.

The study protocol received waivers from the Institute Human Ethics Committee for Postgraduate Research (IHECPGRPHD065) and Institutional Animal Ethics Committee (AIIMS/BPL/IAEC/2020/028), as the study involved neither human participants nor live‐animal experimentation. The Institute Biosafety Committee granted approval for processing the samples (IBSC/AIIMSBhopal/04/2020).

### 2.2. Sample Preparation for *M. tuberculosis* Culture Isolation

All received samples were processed for *Mycobacterium* species isolation following standard protocols, as reported earlier [13]. Briefly, tissue samples were homogenized, decontaminated using the 4% sodium hydroxide–sodium citrate method, and inoculated into MGIT tubes (Becton Dickinson) and incubated in MGIT 960 liquid culture system (Becton Dickinson). Culture‐positive isolates were subjected to molecular identification by Multiplex PCR (mPCR). Among all culture‐positive isolates, six were selected for WGS as described in the study objectives.

### 2.3. Detection of MTBC by Molecular Assays, Including Xpert‐Ultra, Truenat MTB/RIF Dx, and mPCR

The Xpert‐Ultra assay and Truenat MTB/RIF Dx assays were used in the present study to detect MTBC and rifampicin (RIF) resistance in tissue samples according to the standard procedure [13]. mPCR was performed for simultaneous differentiation of MTBC, MAC, and other nontuberculous mycobacteria (NTM) as reported earlier [13, 14].

### 2.4. Drug Susceptibility Testing

Drug susceptibility testing of all MTBC isolates was performed using the GenoType MTBDRplus assay (Hain Life Science, Nehren, Germany) according to the manufacturer′s instructions. The procedure included DNA extraction (using the GenoLyse kit, Hain Life Science, Nehren, Germany, in a BSL‐3 facility), PCR amplification with biotinylated primers, and hybridization in a semiautomated TwinCubator system. Hybridized strips were placed on paper and interpreted based on band patterns in designated zones [13, 15].

### 2.5. Spoligotyping

DNA was extracted from the MTBC culture grown in MGIT tubes by using the CI method, and spoligotyping was done using the commercially available kit (Mapmygenome, Hyderabad, India) to assess the genetic diversity and the MTBC lineages [13].

### 2.6. WGS

WGS was performed on a representative subset of six culture‐positive MTBC isolates. The selection criteria were designed to fulfill three primary objectives: (i) host and tissue diversity, ensuring representation across different animal species (blackbuck, swamp deer, sloth bear, and blue bull) and diverse anatomical sites (both pulmonary and extrapulmonary tissues, including the liver, spleen, heart, and lung lobes); (ii) higher‐resolution genomic characterization, specifically targeting isolates with unresolved, ambiguous, or orphan spoligotyping profiles; and (iii) technical suitability, restricting selection to isolates that yielded sufficient DNA quantity and high molecular quality required for sequencing library preparation.

Briefly, following successful subculture, genomic DNA was extracted from the MTBC isolates for WGS library preparation. DNA was extracted from LJ‐grown MTBC strains using the HiPurA Fast MTB Genomic DNA Purification Kit (Himedia, Cat. No. MB579) according to the manufacturer′s protocol. Briefly, 5 mL of culture suspension, prepared by resuspending two loopfuls of bacterial colonies in phosphate buffer (PB), was centrifuged at 4000 × *g* for 3 min. The pellet was resuspended in 200 *μ*L of additive solution and incubated for 30 min at 37°C with 20 *μ*L of Proteinase K (20 mg/mL). Subsequently, 200 *μ*L of lysis solution was added, and this mixture was incubated at 55°C for 10 min. Following the addition of 200 *μ*L of ethanol (95%–100%), the lysate was transferred to a spin column and centrifuged at 6500 × *g*. The column was washed consecutively with 500 *μ*L of prewash solution and 500 *μ*L of diluted wash solution, followed by centrifugation at maximum speed (16,000 × *g*). DNA was eluted in 200 *μ*L of elution buffer after incubating at room temperature for 1 min. DNA quality was assessed using the Qubit DNA HS Kit (Invitrogen), with acceptable purity defined by OD260/280 ratios of 1.8–2.0 and OD260/230 ratios of 2.0–2.2.

The whole‐genome libraries were prepared according to the manufacturer′s instructions. Briefly, the Ion Plus Fragment DNA Library Prep Kit (Thermo Fisher, Cat. No. 4471252) was used. Genomic DNA was first sheared to ~250 bp fragments using the Ion Shear Plus Kit (Thermo Fisher, Cat. No. 2664777), followed by adapter ligation and barcoding with the Ion Xpress Barcode and Adapters Kit (Thermo Fisher, Cat. No. 4471250). After each step, purification was performed using AMPure beads (Beckman Coulter, Cat. No. A63881). Library quality and concentration were assessed using a 2% E‐gel (Size Select agarose gel) (Thermo Fisher, Cat. No. 2R300723‐01) [16].

Library quantification was performed using the Qubit High‐Sensitivity kit. The libraries were then diluted to a final concentration of 100 pM and subjected to cluster amplification. The libraries were loaded onto 530 chip using the Ion Chef System (Ion‐Torrent/Thermo‐Fisher) and sequenced on the Ion GeneStudio S5 Prime Series System for whole genome sequencing of mycobacteria. Sequencing generated single‐end reads with an average read length of approximately 200 bp. The raw sequencing data obtained from the Ion GeneStudio S5 Plus Prime Series System were further processed and analyzed.

### 2.7. Data Acquisition and Quality Control

Publicly available whole‐genome sequences used in the comparative analysis were downloaded from the NCBI Sequence Read Archive (SRA) using the SRA Toolkit (v2.11.0) (Table S1). The SRA files were converted to FASTQ format using fasterq‐dump. The quality of all raw sequencing reads (both newly sequenced and downloaded) was assessed and processed using fastp. The program was used to trim adapter sequences, and reads with a Phred quality score < 20 was removed. Short reads (< 50 bp) or low‐quality bases (> 30%) were discarded to ensure that only high‐quality data were used for downstream analysis.

### 2.8. Lineage and Drug Resistance Profiling

To determine the MTBC lineage, the quality‐controlled single‐end reads for each isolate were analyzed using TB‐Profiler to screen the reads for known lineage‐defining and drug‐resistance‐conferring mutations against the curated TB‐profiler database.

### 2.9. Reference‐Based Mapping and Coverage Analysis

The high‐quality filtered reads were mapped against the *M. tuberculosis* H37Rv reference genome (accession No. NC_000962.3) and the *M. orygis* reference genome (accession No. CP063804.2) using BWA‐MEM v0.7.17 with default parameters [17]. The resulting SAM files were converted to BAM format, followed by sorting and indexing using SAMtools v1.16.1. Coverage metrics, including the total number of covered bases, percentage of genome covered, and mean sequencing depth were calculated using the coverage function in SAMtools. Variant calling and annotation were performed using SnpEff, and the total number of single‐nucleotide polymorphisms (SNPs) and insertions/deletions (indels) was recorded [18]. These variants were filtered for a minimum read depth 10×, base quality ≥ 20, and a minimum allele frequency of ≥ 90%. The high‐confidence variants were identified using these criteria.

### 2.10. Whole‐Genome SNP Phylogenetic Analysis

To perform a comparative phylogenetic analysis, high‐quality assembled genomes of six study isolates were generated and analyzed along with 133 publicly available full‐length whole‐genome sequences comprising 71 *M. tuberculosis* genomes originating from both animal (wild and domestic) and human hosts across diverse geographic regions, including India, United States, Japan, South Africa, Switzerland, China, South Korea, Peru, Russia, Uganda, Ireland, and Nepal, and an additional 62 *M. orygis* genomes from wild animals, domestic animals, and human hosts from India, Australia, Norway, Canada, and the United States were included in the analysis. Among them, 29 *M. orygis* genomes were derived only from wild and domestic animal hosts, including cattle, from multiple Indian states viz Tamil Nadu, Gujarat, West Bengal, and Madhya Pradesh (Table S1).

Whole‐genome sequences of good quality in terms of sequencing depth, ambiguity, and metadata were chosen for reliable SNP‐based phylogenetic reconstruction. Preference was given to Indian isolates because of their epidemiological relevance, while representative genomes from other regions were included to provide global context. Both human and animal isolates were selected to avoid host bias.

All sequences were recovered from the NCBI SRA database (details of metadata are given in Table S1). A core SNP‐based phylogenetic analysis was performed to infer the evolutionary relationships between the isolates. The Snippy pipeline (v4.6.0) was used for a standardized and reproducible workflow.

The snippy‐core utility was run on the output directories of all samples. This step identified core SNP sites (positions present and varied across all analyzed genomes) and generated a core SNP multiple sequence alignment. Pairwise SNP distances were calculated from the core SNP alignment generated by Snippy using snp‐dists, which computes pairwise distances between all isolate sequences based on variable positions. This distance matrix quantifies genetic divergence among isolates. A maximum likelihood phylogenetic tree was built from the SNP alignment using IQ‐TREE v2.2.6 [19]. The optimal nucleotide substitution model was automatically selected, with the general time reversible model with Gamma‐distributed rate heterogeneity (GTR + G) being chosen. Branch support was robustly assessed using 1000 ultrafast bootstrap replicates (‐bb 1000), with bootstrap values ≥ 95 indicating strong phylogenetic support, 74–94 indicating moderate support, and < 74 indicating weak support. The resulting phylogenetic tree was visualized and annotated using the Interactive Tree Of Life (iTOL v6) to examine evolutionary relationships and identify clusters of closely related strains, with tip labels were cleaned and color‐coded by sample identity and clade to improve clarity and interpretability [20].

The WGS‐based SNP analysis was performed to accurately identify *M. orygis* and differentiate it from other members of the MTBC. For this analysis, six target genes were shortlisted from the literature (i.e., *gyrB*, *Rv2042c*, *PPE55*, *leuS*, *mmpL6*, and *mmpS6*) [4]. Gene sequences were obtained from assembled genomes. Sequence for *gyrB*, *Rv2042c*, *PPE55*, and *leuS* genes was obtained from *M. tuberculosis* H37Rv (accession No. NC_00962.3), and the sequences of *mmpL6* and *mmpS6* genes were recovered from *M. tuberculosis*‐specific deletion one region “TbD1” (accession No. AJ426486.1). Variants were called to identify both known and novel SNPs by annotating the VCF file using the SnpEff tool. The detected mutations were reported for each gene.

### 2.11. Comparative Phylogenetic Analysis of *M. orygis* Genome to Investigate the Host‐Specific Evolution

A phylogenetic analysis was conducted to explore the genetic evolution of the newly sequenced *M. orygis* isolate. For this, the whole‐genome sequence of the studied *M. orygis* animal isolate was compared with publicly available *M. orygis* genomes of human and animal sources to investigate host‐specific genetic evolution. The dataset included 62 *M. orygis* genomes isolated from human and different animal sources across various countries. All sequences were retrieved from the NCBI SRA database (details of metadata are given in Table S1).

## 3. Results

All six MTBC isolates selected for WGS were obtained from four animal host species, including blackbuck (*Antilope cervicapra*, *n* = 2), blue bull (*Boselaphus tragocamelus*, *n* = 2), swamp deer (*Rucervus duvaucelii*, *n* = 1), and sloth bear (*Melursus ursinus*, *n* = 1). The isolates were recovered from both pulmonary and extrapulmonary tissues, including lung lobes, liver, spleen, and heart, suggesting systemic dissemination in some animals (Figure 1). All six culture‐positive isolates were confirmed as MTBC by mPCR, Xpert Ultra, and Truenat MTB assays. Drug susceptibility testing using Line Probe Assay revealed five pan‐susceptible isolates and one isoniazid monoresistant isolate. Spoligotyping identified two orphan isolates, three CAS1‐Delhi lineage isolates, and one unidentified isolate. WGS later reclassified the one orphan isolate as *M. orygis*, and the second orphan isolate as belonging to the Indo‐Oceanic lineage, and confirmed the remaining four isolates, including the previously unidentified strain, as belonging to the CAS1‐Delhi lineage (Tables 1 and 2).

**Figure 1:**
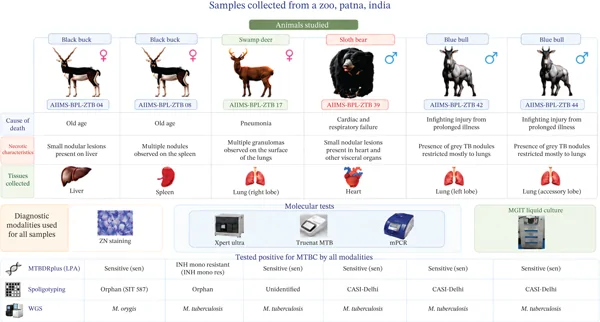
Overview of molecular and phenotypic characteristics of the sequenced isolates.

**Table 1:** Drug susceptibility profile of the samples.

S. no.	TB‐ID	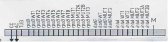	TUB	rpoB WT	rpoB MUT	Kat G WT	Kat G MUT	inhA WT	inhA MUT	RMP	INH	Result
1	AIIMS‐BPL‐ZTB04	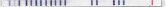	+	+	−	+	−	+	−	S	S	Pan sensitive
2	AIIMS‐BPL‐ZTB08	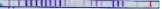	+	+	−	+	−	+	−	S	R	INH monoresistant
3	AIIMS‐BPL‐ZTB17	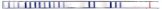	+	+	−	+	−	+	−	S	S	Pan sensitive
4	AIIMS‐BPL‐ZTB39	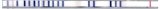	+	+	−	+	−	+	−	S	S	Pan sensitive
5	AIIMS‐BPL‐ZTB42	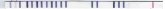	+	+	−	+	−	+	−	S	S	Pan sensitive
6	AIIMS‐BPL‐ZTB44	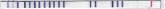	+	+	−	+	−	+	−	S	S	Pan sensitive

*Note:* + = presence of the band; − = absence of the band.

Abbreviations: INH, isoniazid; MUT, mutated; RMP, rifampicin; S, sensitive; WT, wild type.

**Table 2:** Lineage identification of the isolates by spoligotyping.

S. no	TB‐ ID	Spoligotype pattern	Octal number	SIT number	Lineage
1	AIIMS‐BPL‐ZTB04	■■■□□□□□□■■■■□□□□□□□□□□□■■■■■■■■■■□□■■□■■■■	700740007774671	587	Orphan
2	AIIMS‐BPL‐ZTB08	■■■□□□□□■■■■■□□■■■■■■■■■■■■■■■■■■■□□■■□■■■■	701747777774671	NA	Orphan
3	AIIMS‐BPL‐ZTB17	■■■□□□□■■■■■■■■■■■■■■■□□□□□□□□□□□□□□■■■■■■■	703777740000771	357	CAS1‐DELHI
4	AIIMS‐BPL‐ZTB39	■■■□□□□■■■■■■■■■■■■■■■□□□□□□□□□□□□□□■■■■■■■	703777740000771	357	CAS1‐DELHI
5	AIIMS‐BPL‐ZTB42	■■■□□□□■■■■■■■■■■■■■■■□□□□□□□□□□□□□□■■■■■■■	703777740000771	357	CAS1‐DELHI
6	AIIMS‐BPL‐ZTB44	■■■□□□□■■■■■■■■■■■■■■■□□□□□□□□□□□□□□■■■■■■■	703777740000771	357	CAS1‐DELHI

### 3.1. WGS Analysis

WGS of all six isolates yields 293,892–1,175,865 reads per isolate, with 4.31–4.41 Mbp of the genome sequence. The average reference genome coverage across all isolates was 97.51%. The mean sequencing depth across the six isolates was 41.48×, with the lowest depth observed in isolate AIIMS‐BPL‐ZTB08 (21.14×) and the highest in isolate AIIMS‐BPL‐ZTB04 (66.99×) (Table 3).

**Table 3:** Sequencing statistics and genetic variants identified in the sequenced isolates.

S. no.	TB‐ID	Number of reads	Covered bases	Percentage covered	Average depth	No. of SNPs	Indels
1	AIIMS‐BPL‐ZTB04	1175865	4.31 Mbp	98.93%	66.9923	204	26
2	AIIMS‐BPL‐ZTB08	688199	4.41 Mbp	93.65%	21.1417	2114	5578
3	AIIMS‐BPL‐ZTB17	830200	4.41 Mbp	97.39%	32.6896	1813	309
4	AIIMS‐BPL‐ZTB39	293892	4.41 Mbp	98.19%	49.1495	1874	366
5	AIIMS‐BPL‐ZTB42	474271	4.35 Mbp	98.59%	22.8854	1198	92
6	AIIMS‐BPL‐ZTB44	953507	4.34 Mbp	98.29%	55.9968	1274	113

Whole‐genome sequence analysis revealed variability in the number of SNPs and indels among the isolates relative to their respective reference genomes. The isolate AIIMS‐BPL‐ZTB04, identified as *M. orygis*, showed the lowest genomic variation, with 204 SNPs and 26 indels. In contrast, the *M. tuberculosis* isolates demonstrated comparatively higher genomic diversity. Among them, the highest variation was observed in isolate AIIMS‐BPL‐ZTB08, followed by AIIMS‐BPL‐ZTB39 and AIIMS‐BPL‐ZTB17. In contrast, isolates AIIMS‐BPL‐ZTB42 and AIIMS‐BPL‐ZTB44 demonstrated relatively lower variation (Table 3).

A total of 1876 core variable sites were retained for *M. tuberculosis*, compared with 836 core sites for *M. orygis*.

Lineage identification using the TB‐Profiler tool revealed that isolate AIIMS‐BPL‐ZTB04, recovered from the liver tissue of a blackbuck, belonged to *M. orygis* and exhibited gross nodular pulmonary lesions at necropsy. The culture‐positive liver isolate was selected for WGS and was subsequently identified as *M. orygis*. Thus, the sequenced isolate represented an extrapulmonary site in an animal with concurrent pulmonary lesions. The SNP analysis of six target genes (*gyrB,* *Rv2042c*, *PPE55*, *leuS*, *mmpL6*, and *mmpS6*) identified a total of 11 characteristic variants previously reported in the literature, supporting their utility as genetic markers for identification of *M. orygis*. In the gyrB gene, substitutions were observed at positions 5516 (A>G), 6109 (G>A), 6446 (G>T), and 6717 (T>C). The *Rv2042c* gene showed a single nucleotide substitution at position 2288569 (T>G). Two consecutive substitutions were identified in the *PPE55* gene at positions 3751022 (T>G) and 3751023 (C>T). The leuS gene exhibited mutations at positions 44625 (A>T) and 44812 (G>T). Additionally, one SNP each was identified in mmpL6 at position 1220 (T>C) and mmpS6 at position 625 (C>G). These characteristic SNPs supported the identification and differentiation of *M. orygis* from other members of the MTBC complex (Table 4).

**Table 4:** SNPs detected in six targeted genes of *M. orygis* isolates compared with reference genomes.

S. no.	Authors	Host	Gene											References
			*gyrB*				*Rv2042c*	*PPE55*		*leuS*		*mmpL6*	*mmpS6*	
			5516 (A>G)	6109 (G>A)	6446 (G>T)	6717 (T>C)	2288569 (T>G)	3751022 (T>G)	3751023 (C>T)	44625 (A>T)	44812 (G>T)	1220 (T>C)	625 (C>G)	
1	Rani et al., 2025	Human, animal	+	+	+	+	+	+	+	+	+	+	+	[21]
2	Islam et al., 2023	Human	+	−	−	−	−	−	−	+	−	−	+	[4]
3	Napier et al., 2020	Human, animal	−	−	−	−	−	−	−	−	+	−	−	[22]
4	Lipworth, 2019	Human, animal	−	+	−	+	−	−	−	−	−	−	−	[23]
5	Marcos, 2017	Human	−	+	−	−	−	−	−	−	−	−	−	[24]
6	Thapa et al., 2016	Animal	−	−	+	−	−	−	−	−	−	−	−	[25]
7	Thapa et al., 2015	Animal	−	+	−	−	−	+	+	−	−	−	−	[26]
8	Ingen et al., 2012	Human, animal	−	−	−	−	+	−	−	−	−	−	−	[27]
9	Huard et al., 2006	Human, animal	−	+	−	−	−	+	+	−	−	−	−	[28]
10	This study	Animal	+	+	+	+	+	+	+	+	+	+	+	NA

The AIIMS‐BPL‐ZTB08 isolate was identified as *M. tuberculosis* Lineage 1 (Indo‐Oceanic), whereas the remaining four isolates belonged to *M. tuberculosis* Lineage 3 (CAS1‐Delhi). The sequencing quality metrics and genome coverage obtained for all isolates were adequate for downstream comparative genomic and phylogenetic analyses. All six genomes generated in this study were submitted to the NCBI SRA database under the accession No. SRR36284444, SRR36284445, SRR36284446, SRR38817758, SRR38817759, and SRR38817760 under the BioProject number PRJNA1372978.

### 3.2. Whole‐Genome SNP Phylogenetic Analysis of *M. orygis* and *M. tuberculosis* Isolates

A whole‐genome SNP‐based phylogenetic analysis showed that the AIIMS‐BPL‐ZTB04 (*M. orygis*) isolate showed the closest phylogenetic relatedness to a cattle‐derived *M. orygis* isolated from West Bengal (SRR10251188) (Figure 2). To investigate the host‐specific evolution, when a comparative phylogenetic analysis was conducted that included publicly available *M. orygis* genomes from animals as well as human hosts. In this analysis, this isolate clustered closely with human‐origin *M. orygis* isolated from a patient in Karnataka, India (SRR10321143), and Jharkhand, India (SRR10321134). Pairwise SNP distance analysis exhibited a 7 SNP difference with both isolates, supported by strong bootstrap values of 97 and 100, respectively, indicating a close genomic relationship.

**Figure 2:**
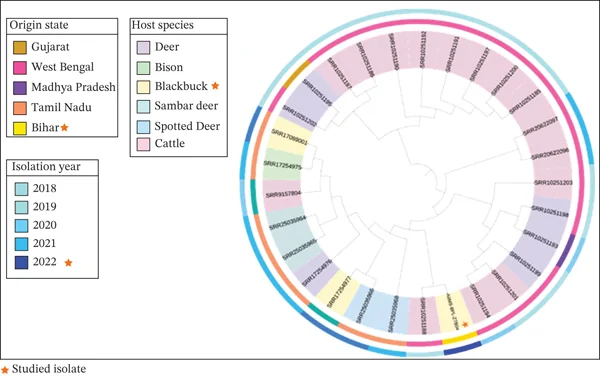
Phylogenetic analysis of the newly sequenced *M. orygis* isolate with publicly available *M. orygis* isolates from India. (The outermost ring represents the year of isolation, the inner ring indicates the state of isolation, and different host species are depicted using distinct colors).

In contrast, the isolate differed by 28 SNPs from an Indian cattle‐derived genome (SRR10251188), and had a bootstrap support of 93, suggesting relatively greater genetic divergence (Table S2). These findings reflect shared lineage relationships across host‐associated isolates within India and may indicate potential cross‐host circulation; however, these observations are preliminary and do not imply interspecies transmission (Figure 3).

**Figure 3:**
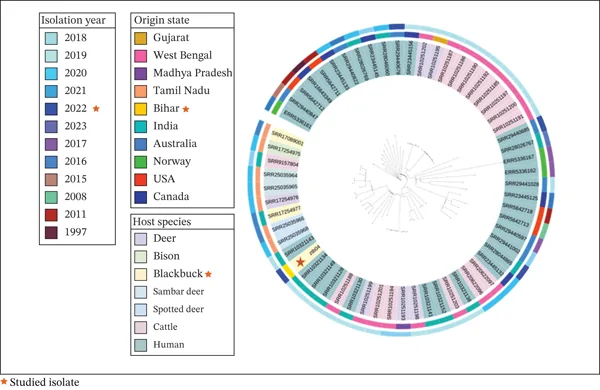
Phylogenetic analysis of the newly sequenced *M. orygis* isolate with publicly available *M. orygis* isolates of animal and human origin around the globe. (The outermost ring represents the year of isolation, the inner ring indicates the country and state of isolation, and different host species are depicted using distinct colors).

#### 3.2.1. *M. tuberculosis*

The whole‐genome SNP‐based phylogenetic analysis revealed that all five *M. tuberculosis* study isolates clustered closely with previously reported MTBC genomes from India and other geographical regions. The AIIMS‐BPL‐ZTB08 showed close relatedness to an Indian cattle isolate (SRR8375622; with a pairwise distance of 78 SNPs distance; bootstrap support, 87) and an even closer relationship to another Indian cattle isolate (SRR29322291; with a pairwise distance of 61 SNPs distance). Its distance from the reference genome was 206 SNPs (bootstrap support, 100). The AIIMS‐BPL‐ZTB17 clustered closely with an Indian human isolate (SRR15506888; with a pairwise distance of 38 SNPs distance; bootstrap support, 88) and showed high similarity to AIIMS‐BPL‐ZTB39 (with a pairwise distance of 25 SNPs distance; bootstrap support, 98). Its distance from the reference genome was 197 SNPs (bootstrap support, 100).

The AIIMS‐BPL‐ZTB42 and AIIMS‐BPL‐ZTB44 strains isolated from different body sites in the same host exhibited 99.73% genomic similarity and complete genetic identity (with 0 SNPs differences; bootstrap support, 100), suggesting dissemination of same strain to various organs. They clustered closely with SRR15506888 (with 38 SNPs distance; bootstrap support, 88), AIIMS‐BPL‐ZTB17 (with 33 SNPs distance; bootstrap support, 98), and another human‐derived isolate (SRR34635839; with 46 SNPs distance; bootstrap support, 62). Both differed from the reference genome by 195 SNPs distance (bootstrap support, 100) (Table S2). This clustering reflects close genomic resemblance between the study isolates and MTBC strains derived from geographically and host‐diverse sources (Figure 4).

**Figure 4:**
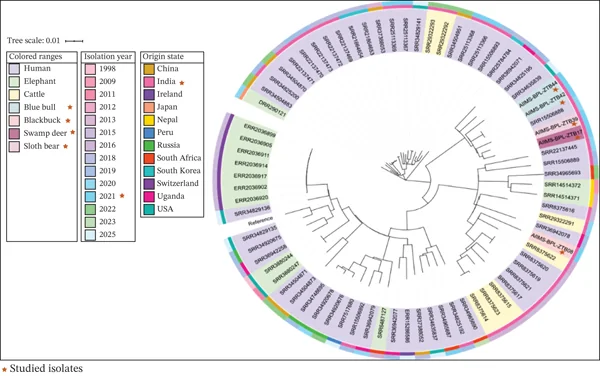
Phylogenetic analysis of the newly sequenced *M. tuberculosis* isolates with publicly available *M. tuberculosis* isolates from India and around the globe. (The outermost ring represents the year of isolation, the inner ring indicates the country of isolation, and different host species are depicted using distinct colors).

## 4. Discussion

Although MTBC species are primarily adapted to humans, they are capable of causing chronic and devastating infections across a broad range of domestic and wild animals. Infected animals can act as reservoir hosts, presenting substantial challenges to disease control and eradication efforts [29]. The findings of the present study are consistent with earlier reports documenting considerable TB burden among captive zoo animals, with potential for fatal outcomes [30].

Given the global rise of drug‐resistant TB and its complex challenges at the human‐animal interface, monitoring resistance profiles in wildlife is essential for both veterinary management and epidemiological tracking [31]. Five pan‐susceptible strains and one isolate with resistance to isoniazid were found in our study, while no multiresistant (MDR) strains were reported. This represents a resistance profile limited to our cohort, and hence any broad estimation of drug‐resistance prevalence across captive wildlife populations cannot be made. Highlighting the importance of conducting surveillance on a larger scale and across multiple hosts in the future.

In this study, WGS and phylogenetic analysis of sequenced *M. orygis* and *M. tuberculosis* isolates provided markedly higher resolution than the traditional molecular typing methods employed in our previous study [13]. Six representative culture‐positive isolates were selected to capture lineage diversity, resolve ambiguous genotyping results, and investigate within‐host genomic variation across different animal hosts and anatomical sites.

Both blackbuck cases presented with extrapulmonary TB involving the liver and spleen, compatible disseminated disease. In the first animal, small nodular hepatic lesions were observed; spoligotyping classified the isolate as an orphan strain (SIT 587), whereas WGS reclassified it as *M. orygis*. Hepatic granulomas are consistent with chronic hematogenous dissemination and have been previously described in wild ungulates [5, 30].

### 4.1. Blackbuck

AIIMS‐BPL‐ZTB04, identified as *M. orygis* was recovered from liver tissue of a blackbuck with concurrent gross pulmonary nodular lesions. This finding should be viewed in the context of disseminated pulmonary infection. Despite lungs being the primary filter of inhaled mycobacteria, the pathogen spreads to other organs through hematogenous route and can settle in any organs such as lymph nodes, pleura, kidneys, and liver, involving only that organ. However, often when the indigenous and repeated mycobacteraemia from the pulmonary lesions may lead to dissemination to other organs along with pulmonary TB. This form is known as disseminated TB and is most serious form of the disease [30]. The extrapulmonary involvement of TB has been reported in Indian animals with *M. orygis* infection, involving liver and other organs [32]. In the second blackbuck case, multiple splenic nodules were detected; although spoligotyping results identified it as an orphan without SIT assignment WGS classified the isolate within the Indo‐Oceanic lineage. This discordance between spoligotyping and WGS outcomes likely reflects the limited discriminatory resolution of spoligotyping, particularly for less common MTBC members and atypical lineages. Splenic involvement may be compatible with systemic spread via hematogenous or lymphatic routes, and recovery of MTBC from splenic tissue has been reported previously in wildlife [33]. The Indo‐Oceanic lineage is commonly associated with human TB in the Indian subcontinent [34], and its detection in this animal raises the possibility of exposure to strains circulating in human or livestock populations; however, the source of infection cannot be determined from the present data. The identification of two distinct MTBC lineages across the two blackbuck cases underscores the complex epidemiology of TB in captive wildlife and may reflect exposure to multiple infection sources. Both affected animals were aged females, indicating that age‐associated immunosenescence in ungulates may have predisposed them to chronic infection.

### 4.2. Swamp Deer

The swamp deer exhibited multiple granulomatous lesions on the lung surface, consistent with chronic pulmonary TB following respiratory exposure. Pulmonary localization is commonly reported in cervids and may reflect respiratory exposure under captive conditions [35]. Although spoligotyping failed to resolve the isolate, WGS classified it within the CAS1‐Delhi lineage. The animal died following prolonged illness and infighting injury, suggesting that chronic TB may have compromised its overall health, potentially acting as a predisposing rather than direct cause of death.

### 4.3. Sloth Bear

The sloth bear showed evidence of disseminated TB with nodular lesions involving the heart and other visceral organs. Cardiac involvement is uncommon in TB and typically indicates advanced systemic disease [30]. Both spoligotyping and WGS consistently identified the isolate as CAS1‐Delhi lineage, a lineage widely associated with human and livestock‐associated TB in India. The multiorgan dissemination pattern, including cardiac involvement, suggests that advanced systemic TB contributed to cardiorespiratory failure. The identification of a human‐associated lineage is compatible with the possibility of interspecies transmission at the wildlife–livestock–human interface; however, lineage assignment alone cannot establish the source of infection or interspecies transmission.

### 4.4. Blue Bull

The blue bull presented with pulmonary TB characterized by gray nodular lesions predominantly confined to the lungs, consistent with pulmonary involvement. Two isolates recovered from distinct anatomical sites within the same animal, the left and accessory lung lobes, were concordantly classified as CAS1‐Delhi lineage by both spoligotyping and WGS. The absence of SNP difference between these isolates is consistent with infection by the same or a highly similar strain (Figure 1 and Table 2).

### 4.5. *M. orygis*: Reclassification and Host Range

The reclassification of the SIT 587 orphan isolate as *M. orygis* by WGS is a finding of considerable significance and is consistent with prior observations of spoligotype‐based misclassification within MTBC databases [25, 27]. This supports the greater discriminatory capacity of WGS for accurate species‐level characterization within the MTBC, particularly for less‐studied members such as *M. orygis*.

*M. orygis* was originally described by Ingen et al. [27], as an MTBC member isolated from animals of the Bovidae family, including oryxes, deer, gazelles, antelopes, and waterbucks, though its primary reservoir host remains to be determined. Subsequent reports have substantially expanded its known host range. Duffy et al. [36] demonstrated its involvement in human TB, supporting its zoonotic potential, and more recent studies have confirmed infection across a wide range of wildlife hosts, including deer, rhinoceros, bison, blue bull, and blackbuck [5, 26, 27, 37]. In the present study, *M. orygis* was isolated from the liver tissue of a blackbuck (*Bovidae* family), further contributing to the documented host range of this species and reinforcing the need for its inclusion in TB surveillance programs across livestock and wildlife populations, particularly in the context of zoonotic risk.

### 4.6. Phylogenomic Analysis

The whole‐genome SNP‐based phylogenetic analysis conducted in this study provides insights into the evolutionary dynamics and genetic diversity of the sequenced isolates relative to publicly available genomes from human and animal hosts across India and other countries. Prior reports of *M. orygis* from eastern India, including Kolkata, have documented *M. orygis* in cattle [38], suggesting broader circulation of this organism across regional livestock populations.

Phylogenetic analysis of the six isolates, including five *M. tuberculosis* and one *M. orygis* isolate, demonstrated clustering of all study isolates with previously reported MTBC genomes from India and other geographic regions, indicating genomic relatedness among isolates obtained from diverse host populations. These findings demonstrate genetic relatedness between the animal‐derived isolates and previously reported cattle and human‐derived isolates.

Recent reports from India have documented *M. tuberculosis* in cattle, raising the possibility of human‐to‐livestock spillover [31, 39]. Similarly, reverse zoonotic transmission has been proposed in the detection of *M. tuberculosis* in elephants [40, 41]. Collectively, these observations point to the complex host ecology of MTBC and are suggestive of cross‐host circulation of genetically related strains, though definitive conclusions regarding transmission directionality cannot be drawn without supporting epidemiological data.

Studies of *M. orygis*‐specific SNPs across six targeted gene loci, *gyrB*, *Rv2042c*, *PPE55*, *leuS*, *mmpL6*, and *mmpS6*, have shown considerable variability in prior studies, with SNP patterns often inconsistent across investigations (summarized in Table 4). In contrast, the present study identified all 11 SNPs across all six gene loci, yielding a comprehensive and internally consistent genomic profile reported, consistent with the findings of Rani et al. [21]. This complete SNP resolution provides strong genomic evidence supporting the identification of *M. orygis* in animal hosts and further underscores the critical importance of WGS for precise species‐level characterization within the MTBC. The occurrence of *M. tuberculosis* Lineages 1 (Indo‐Oceanic) and 3 (CAS1‐Delhi) in captive wildlife highlights the importance of the human–animal interface in TB surveillance and supports consideration of animal and occupational exposure histories in pulmonary TB risk assessment.

The study also highlights the utility of WGS‐based One Health TB surveillance for accurate species identification, lineage tracing, and assessment of genomic relatedness. Such approaches will strengthen targeted TB control strategies aligned with the goals of the End TB Strategy and SDG 3.3. Further longitudinal multihost studies integrating genomic and epidemiological data are needed to clarify the epidemiological significance of these genomic relationships and investigate potential transmission pathways in India [42].

## 5. Limitations of the Study

The extent to which the findings can be generalized is constrained by the limited sample size and selection sampling. Only six isolates from different organs across multiple animal hosts were subjected to WGS. These six culture‐positive isolates were selected to capture distinct genomic and epidemiological contexts within the study. Selection was further constrained by technical suitability for sequencing, and only isolates demonstrating successful subculture, pure growth, and sufficient quantities of high‐quality DNA for library preparation were processed for WGS. An additional limitation was the absence of multidrug‐resistant isolates and other MTBC species, which limited the breadth of genomic comparisons.

Furthermore, the absence of concurrent sampling of livestock, humans, or environmental sources from the vicinity of the sequenced cases, along with limited information on housing conditions of animals, shared environments, and human or livestock contact, restricts the ability to assess local epidemiological linkage or potential cross‐host transmission. Future studies incorporating larger, longitudinal, and multihost sampling, combined with higher sequencing depth, standardized phenotypic characterization, and integrated One Health epidemiological frameworks, will be essential to validate and expand these preliminary observations.

## 6. Conclusion

This study demonstrates the usefulness of WGS application and phylogenomic approach in determining the correct lineages and genomic diversity among the MTBC in captive wild animals. The WGS could identify the unassigned or misclassified “orphan” profiles, as *M. orygis*. The comparative phylogenomic revealed that this *M. orygis* shared genomic structure with other sequenced isolates from cattle and humans of India and other parts of the world, highlighting potential cross‐host genetic links. The characterization of the *M. tuberculosis* isolates also confirmed the presence of two predominantly human lineages namely Lineage 1 (Indo‐Oceanic) and Lineage 3 (CAS1‐Delhi) with other human and livestock isolates. These findings underscore that captive wild animals can harbor both specialized animal‐associated pathogens (*M. orygis*) as well as human‐adapted strains of *M. tuberculosis*, presenting a challenge to national and global TB elimination programs.

## Funding

No funding was received for this manuscript.

## Conflicts of Interest

The authors declare no conflicts of interest.

## Supporting Information

Additional supporting information can be found online in the Supporting Information section.

## Supporting information


**Supporting Information 1** Table S1: Detailed information.

**Supporting Information 2** Table S2: Core SNP differences and bootstrap values between the studied isolates.

## Data Availability

All six genomes generated in this study were submitted to the NCBI SRA database under the accession No. SRR36284444, SRR36284445, SRR36284446, SRR38817758, SRR38817759, and SRR38817760, under the BioProject number PRJNA1372978.
